# A game theoretic framework for incentive-based models of intrinsic motivation in artificial systems

**DOI:** 10.3389/fpsyg.2013.00791

**Published:** 2013-10-30

**Authors:** Kathryn E. Merrick, Kamran Shafi

**Affiliations:** School of Engineering and Information Technology, University of New South Wales, Australian Defence Force AcademyCanberra, ACT, Australia

**Keywords:** intrinsic motivation, game theory, agents, prisoner's dilemma, leader, chicken, battle of the sexes

## Abstract

An emerging body of research is focusing on understanding and building artificial systems that can achieve open-ended development influenced by intrinsic motivations. In particular, research in robotics and machine learning is yielding systems and algorithms with increasing capacity for self-directed learning and autonomy. Traditional software architectures and algorithms are being augmented with intrinsic motivations to drive cumulative acquisition of knowledge and skills. Intrinsic motivations have recently been considered in reinforcement learning, active learning and supervised learning settings among others. This paper considers game theory as a novel setting for intrinsic motivation. A game theoretic framework for intrinsic motivation is formulated by introducing the concept of optimally motivating incentive as a lens through which players perceive a game. Transformations of four well-known mixed-motive games are presented to demonstrate the perceived games when players' optimally motivating incentive falls in three cases corresponding to strong power, affiliation and achievement motivation. We use agent-based simulations to demonstrate that players with different optimally motivating incentive act differently as a result of their altered perception of the game. We discuss the implications of these results both for modeling human behavior and for designing artificial agents or robots.

## Introduction

Game theory is the study of strategic decision-making (Guillermo, 1995). It has been used to study a variety of human and animal behaviors in economics, political science, psychology, biology, and other areas. Game theoretic approaches have also been utilized in robotics for tasks such as multi-robot coordination and optimization (Meng, 2008; Kaminka et al., 2010) as well as for analyzing and implementing behavior in software agents (Parsons and Wooldridge, 2002). This paper presents a game theoretic framework for intrinsic motivation and considers how motivation might drive cultural learning during strategic interactions. The work provides stepping stones toward intrinsically motivated, game theoretic approaches to modeling strategic interactions. Potential applications include the study of human behavior or modeling open-ended development in robots or artificial agents.

In humans, individual differences in the strength of motives such as power, achievement and affiliation have been shown to have a significant impact on behavior in social dilemma games (Terhune, 1968; Kuhlman and Marshello, 1975; Kuhlman and Wimberley, 1976; Van Run and Liebrand, 1985) and during other kinds of strategic interactions (Atkinson and Litwin, 1960). Some models of these phenomena exist for artificial agents (Simkins et al., 2010; Merrick and Shafi, 2011), but these models have not yet been widely studied for strategic interactions, competition and cooperation between artificial agents.

This paper presents a game theoretic approach to modeling differences in decision-making between individuals caused by differences in their perception of the payoff during certain strategic interactions. Specifically we consider cases where differences in perception are caused by different motivational preferences held by individuals. We study strategic decision-making in the context of mixed-motive games. Four archetypical two-by-two mixed-motive games are considered: prisoner's dilemma (PD), leader, chicken, and battle-of-the-sexes (BoS) (Rapoport, 1967; Colman, 1982). We introduce the concept of optimally motivating incentive and demonstrate that agents with different optimally motivating incentives perceive the four games differently. We show that the perceived games have different Nash Equilibrium (NE) points (Nash, 1950) to the original games. This causes agents with different optimally motivating incentives to act differently. We discuss the implications of these results both for modeling human behavior and for designing artificial agents or robots with certain behavioral characteristics.

In the remainder of this Section, section Mixed-Motive Games introduces mixed-motive games and section Solution Strategies for Mixed-Motive Games reviews relevant existing models of strategic decision-making. Section Solution Strategies for Mixed-Motive Games also discusses the specific contributions of this paper in that context and introduces the background formal notations used in the rest of the paper. Section Incentive-Based Models of Motivation reviews literature from motivational psychology about the influence of incentive-based motivation on decision-making as inspiration for the new models in sections Materials and Methods. Sections Materials and Methods introduces our new notation for incentives and shows how each of the four mixed-motive games are transformed into various new games when different optimally motivating incentives are chosen for agent players. Section Results presents a suite of agent-based simulations demonstrating that players with different optimally motivating incentive act differently as a result of their altered perception of the game. We conclude in section Discussion with a discussion of the implications of the work and future directions it may take.

### Mixed-motive games

This paper will consider two-player mixed motive games with the generic structure shown in Matrix 1. Each player, (Player 1 and Player 2) has a choice of two actions: *C* or *D*. Depending on the combination of actions chosen by both players, Player 1 is assigned a payoff value *V*_1_ and Player 2 is assigned a payoff value *V*_2_. *V*_1_ and *V*_2_ can have values of *T*, *R*, *P*, or *S*. The value *R* is the reward if both players choose *C*. In other words, *R* is the reward for a (*C*, *C*) outcome. *P* is the punishment if both players defect [joint *D* choices leading to a (*D*, *D*) outcome]. In a mixed-motive game, *P* must be less than *R*. *T* represents the temptation to defect (choose action *D*) from the (*C*, *C*) outcome and thus, in a mixed-motive game *T* must be greater than *R*. Finally, *S* is the sucker's payoff for choosing *C* when the other player chooses *D.*

Formally, the game **G** presents players with a payoff matrix:

$$G=\left[\begin{array}{cc} P & T\\ S & R \end{array}\right]$$

The generic game **G** can be used to define a number of specific games by fixing the relationships between *T, R*, *P*, and *S*. Four well-known two-by-two mixed motive games and the relationships that define them are (Colman, 1982):

1. Prisoner's Dilemma: *T* > *R* > *P* > *S*
2. Leader: *T* > *S* > *R* > *P*
3. Chicken: *T* > *R* > *S* > *P*
4. Battle of the Sexes: *S* > *T* > *R* > *P*

A number of variations of these games do exist (as well as other distinct games), but this paper will focus on the four games as defined above.

Matrix 1. A generic two-by-two mixed-motive game **G**. *T* must be greater than *R* and *R* must be greater than *P*.

**Table:** 

	Player 2		
Player 1		*D*	*C*
	*D*	*P, P*	*T, S*
	*C*	*S, T*	*R, R*

The PD game (Rapoport and Chammah, 1965; Poundstone, 1992) is perhaps the most well-known of the four games studied in this paper. It derives its name from a hypothetical strategic interaction in which two people are arrested for involvement in a crime. They are held in separate cells and cannot communicate with each other. The police have insufficient evidence for a conviction unless at least one of the prisoners discloses certain incriminating information. Each prisoner has a choice between concealing information from the police (action *C*) or disclosing it (action *D*). If both conceal, both with be acquitted and the payoff to both will be *V*_1_ = *V*_2_ = *R*. If both disclose, both will be convicted and receive minor punishments: *V*_1_ = *V*_2_ = *P*. If only one prisoner discloses information he will be acquitted and, in addition, receive a reward for his information. In this case, the prisoner who conceals information will receive a heavy punishment. For example if Player 1 discloses and Player 2 conceals, the payoffs will be *V*_1_ = *T* and *V*_2_ = *S*. Player 2 in this situation is sometimes referred to as the “martyr” because he generates the highest payoff for the other player and the lowest payoff for himself.

The PD game has been used as a model for arms races, voluntary wage restraint, conservation of scarce resources and the iconic “tragedy of the commons” (see Colman, 1982, for a review). More recently, however, biologists have argued that individual variation in motivation and perception means that a majority of strategic interactions do not, in fact, conform to the PD model (Johnson et al., 2002). The models presented in our paper demonstrate one possible explanation for this latter view. Specifically, they show how a valid PD matrix can be transformed into another game that no longer represents a PD scenario as a result of individuals having different motives.

The game of Leader (Rapoport, 1967) is an analogy for real-world interactions such as those between pedestrians or drivers in traffic. For example, suppose two pedestrians wish to enter a turnstile. Each must decide whether to walk into the turnstile first (action *D*) or concede right of way and wait for the other to walk in (action *C*). If both pedestrians wait, then both will be delayed and receive payoffs *V*_1_ = *V*_2_ = *R*. If they both decide to walk first, a socially awkward situation results in the worst payoff *V*_1_ = *V*_2_ = *P* to both. If one decides to walk and the other waits, the “leader” will be able to walk through unimpeded, receiving the highest payoff *T*, while the “follower” will be able to walk through afterwards giving the second best payoff *S*. Other examples of real world interactions abstracted by the Leader game include two drivers at opposite ends of a narrow, one-lane bridge, or two drivers about to merge from two lanes into one. In some such real-world situations there are rules of thumb that prevent the leader game from emerging, for example flashing headlights at a bridge to concede right of way. However, when such communication fails or is impossible, individuals' motivations have an influential role in decision-making and in how individuals interpret the scenario. We make the standard assumption that there is no communication between agents.

In the game of Chicken two motorists speed toward each other on a collision course. Each has the option of swerving to avoid a collision, and thereby showing themselves to be “chicken” (action *C*) or of driving straight ahead (action *D*). If both players are “chicken,” each gets a payoff of *V*_1_ = *V*_2_ = *R*. If only one player is “chicken” and the other drives straight on, then the “chicken” loses face and the other player, the “exploiter,” wins a prestige victory. For example if Player 1 is “chicken” and Player 2 drives, the payoffs will be *V*_1_ = *S* and *V*_2_ = *T*. If both players drive a collision will occur and both players will receive the worst payoff *V*_1_ = *V*_2_ = *P*. The game of Chicken has also been used to model real-world scenarios in national and international politics involving bilateral threats, as well as animal conflicts and Darwinian selection of evolutionarily stable strategies (Maynard-Smith, 1982).

Finally, the BoS game can be thought of as modeling a predicament between two friends with different interests in entertainment. Each prefers a certain form of entertainment that is different to the other, but both would rather go out together than alone. If both opt for their preferred entertainment, leading to a (*C, C*) outcome, then each ends up going alone and receiving a payoff of *V*_1_ = *V*_2_ = *R*. A worse outcome (*D, D*) results if both make the sacrifice of going to the entertainments they dislike as they both end up alone and *V*_1_ = *V*_2_ = *P*. If, however, one chooses their preferred entertainment and the other plays the role of “hero” and makes the sacrifice of attending the entertainment they dislike then the outcome is better for both of them (either *V*_1_ = *T* and *V*_2_ = *S* or *V*_1_ = *S* and *V*_2_ = *T*). The payoff matrix for BoS is relatively similar to that of Leader, with the only difference in the definition being the relationship between *T* and *S*. In Leader *T* > *S*, while in BoS *S* > *T*. This reflects the real-world relationship that is often perceived between leadership and sacrifice (Van Knippenberg and Van Knippenberg, 2005). We will see in section Results that some of the game transformations that are perceived by agents using our model of optimally motivating incentive also reflect this relationship.

### Solution strategies for mixed-motive games

A strategy σ is a function that takes a game as input and outputs an action to perform according to some plan of play. This paper will focus on pure strategies, such as “always choose action *C*” and mixed strategies that make a stochastic choice between two pure strategies with a fixed frequency. Suppose we denote the probability that Player 2 will choose action *C* as *P*_2_(*C*), then the expected payoff for the two pure strategies available to Player 1 (“always play *C*” or “always play *D*”) can be computed as follows:

$$\begin{array}{l} E_{1}(C)=P_{2}(C)R+[1-P_{2}(C)]S\\ E_{1}(D)=P_{2}(C)T+[1-P_{2}(C)]P \end{array}$$

Using this information, a player can choose the strategy with the maximum expected payoff. A variation on this idea that takes into account individual differences in preference is utility theory (Keeney and Raiffa, 1976; Glimcher, 2011). Utility theory acknowledges that the values of different outcomes for different people are not necessarily equivalent to their raw payoff values *V.* Formally, a utility function *U*(*V*) is a twice differentiable function defined for *V* > 0 which has the properties of non-satiation [the first derivative *U*′(*V*) > 0] and risk aversion [the second derivative *U*″(*V*) < 0]. The non-satiation property implies that the utility function is monotonic, while the risk aversion property implies that it is concave. Utility theories were first proposed in the 1700s and have been developed and critiqued in a range of fields including economics (Kahneman and Tversky, 1979) and game theory (Von Neumann and Morgenstern, 1953).

Alternatives have also been proposed to model effects that are inconsistent to utility theory. Examples include prospect theory (Kahneman and Tversky, 1979) and lexicographic preferences (Fishburn, 1974). The models in this paper can also be thought of as an alternative to utility theory that uses theories of motivation to determine how to compute individuals' preferences. Various other techniques have been proposed to model decision-making under uncertainty, that is, when it is not possible to assign meaningful probabilities to alternative outcomes. Many of these techniques capture “rules of thumb” or heuristics used in human decision-making (Gigerenzer and Todd, 1999). Examples include the maximax, maximin, and regret principles.

The strategies chosen by players and their corresponding payoffs constitute a NE (Nash, 1950) if no player can benefit by changing their strategy while the other player keeps theirs unchanged. This latter definition covers mixed strategies *M* in which players make probabilistic random choices between actions. Formally, if we consider a pair of strategies, σ_1_ and σ_2_, and denote the expected payoff for Player 1 using σ_1_ against Player 2 using σ_2_ as *E*_1_(σ_1_, σ_2_), then the two strategies are in equilibrium if *E*_1_(σ_1_, σ_2_) ≥ *E*_1_(σ′_1_, σ_2_) for all σ′_1_ ≠ σ_1_. In other words, the strategies are in equilibrium if there is no alternative strategy for Player 1 that would improve Player 1's expected payoff against Player 2 if Player 2 continues to use strategy σ_2_ (Guillermo, 1995).

**Figure 1:**
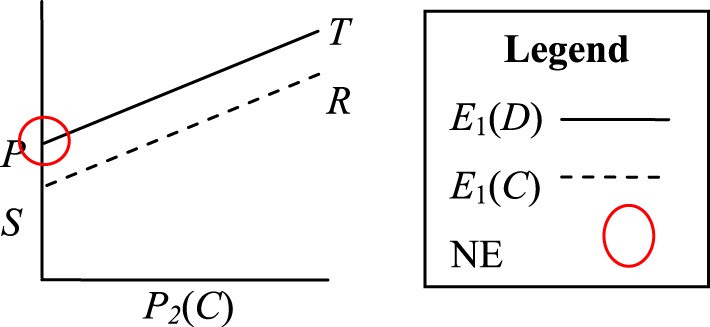
**Visualization of the Prisoner's Dilemma payoff structure *T* > *R* > *P* > *S***. The Nash Equilibrium (NE) is circled.

Suppose we consider the principles discussed above with reference to the four games described in section Mixed-Motive Games. In the PD game there is a pure strategy equilibrium point (*D*, *D*) from which neither player benefits from unilateral deviation, although both benefit from joint deviation. We can visualize this game in terms of expected payoff as shown in Figure 1. We denote the probability of Player 2 choosing *C* as *P*_2_(*C*), the expected payoff if Player 1 chooses *D* as *E*_1_(*D*), and the expected payoff for Player 1 choosing *C* as *E*_1_(*C*). The visualization shows that the definition of PD (*T* > *R* > *P* > *S*) implies that *E*_1_(*D*) > *E*_1_(*C*) regardless of *P*_2_(*C*). In other words, the strategy of choosing *D* dominates the strategy of choosing *C*. The NE for this game (*D*, *D*) is shown circled in Figure 1.

**Figure 2:**
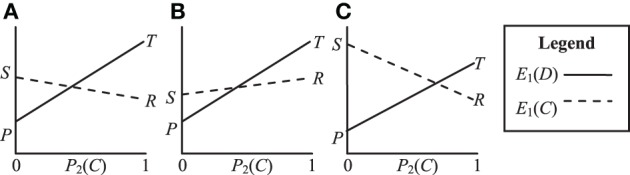
**Visualization of the payoff structures for (A) Leader *T* > *S* > *R* > *P*, (B) Chicken *T* > *R* > *S* > *P* and (C) Battle of the Sexes *S* > *T* > *R* > *P***.

In contrast to the PD game, the Leader, Chicken and BoS games all have *E*_1_(*D*) > *E*_1_(*C*) for *P*_2_(*C*) = 1 and *E*_1_(*D*) < *E*_1_(*C*) for *P*_2_(*C*) = 0. In other words, these games have two asymmetric equilibrium points (*C*, *D*) and (*D*, *C*). However, neither of these equilibrium points is strongly stable because the players disagree about which is preferable. The three games do, however, have a mixed-strategy NE, meaning that players will tend to evolve strategies that choose *C* with some fixed probability. We can also visualize these games in terms of their expected payoff as shown in Figure 2. The NE probability of players choosing *C* is defined by the point at which *E*_1_(*D*) and *E*_1_(*C*) intersect, i.e.:

$$\begin{array}{l} \;E_{1}(C)=E_{1}(D)\\ {}[R-S]P_{2}(C)+S=[T-P]P_{2}(C)+P\\ \;P_{2}(C)=\frac{P-S}{R-S-T+P} \end{array}$$

and likewise for *P*_1_(*C*).

Evolutionary game theory (Maynard-Smith, 1982) combines classical game theory with learning. Evolutionary dynamics predict the equilibrium outcomes of a multi-agent system when the individual agents use learning algorithms to choose actions in iterative game-play. Two-population replicator dynamics, for example, model learning when players may have different strategies. In this model, suppose we combine the probabilities of Player 1 playing *C* and *D* in a vector form *p* = [*p*_*C*_, *p*_*D*_] such that *p*_C_ = *P*_1_(*C*) and *p*_D_ = *P*_1_(*D*) and the probabilities of Player 2 playing *C* and *D q* = [*q*_*C*_, *q*_*D*_] such that *q*_*C*_ = *P*_2_(*C*) and *q*_*D*_ = *P*_2_(*D*). The replicator dynamics in this case are:

(1) $$\Delta p_{i}=p_{i}[{\left(Gq\right)}_{i}-pGq^{T}]$$

(2) $$\Delta q_{i}=q_{i}[{\left(pG^{T}\right)}_{i}-pG^{T}q^{T}]$$

where **G** is the payoff matrix defined by the game being played. In this model, pure strategies tend to dominate over time and mixed-strategies are unstable.

In this paper, we use two-population replicator dynamics to model cultural learning (as opposed to biological evolution) when mixed-motive games are played iteratively. Borgers and Sarin (1997) showed that Cross' learning model for two players iteratively playing “habit forming games” converges to asymmetric continuous time replicator dynamics. Our approach is a stepping-stone toward simulating and analyzing strategic interactions between agents modeling known motive profiles.

While classical game theory discussed above offers a wide range of insights into behavior in strategic interactions, it is not necessarily designed to model human decision-making. In fact, there is evidence of humans not conforming to NE strategies in many kinds of strategic interaction (Terhune, 1968; McKelvey and Palfrey, 1992; Li et al., 2010). As a result, researchers have started to develop alternative approaches. The field of behavioral game theory (Camerer, 2003, 2004) is concerned with developing models of behavior under assumptions of bounded rationality. These models take into account factors such as the heterogeneity of a population, the ability of individuals to learn and adapt during strategic interactions and the role of emotional and psychological factors in strategic decision-making. The purposes of this work fall into two broad categories: (1) to produce computational models that can explain and predict human behavior during strategic interactions that does not conform to classical game theoretic models (Valluri, 2006) and (2) to build artificial systems that can exhibit certain desirable behavioral characteristics such as cooperation or competitiveness (Sandholm and Crites, 1996; Claus and Boutilier, 1998; Vassiliades and Christodoulou, 2010), cooperation during strategic interactions (Valluri, 2006) and improved performance against human adversaries who also have bounded rationality and limited observation (Pita et al., 2010). The work in our paper differs from previous work in this area by its focus on the role of motivation in decision-making.

### Incentive-based models of motivation

In motivational psychology, incentive is defined as a situational characteristic associated with possible satisfaction of a motive (Heckhausen and Heckhausen, 2008). A range of incentive-based motivation theories exist, dealing with both internal and external incentives. Examples of internal incentives include the novelty, difficulty or complexity of a situation. Examples of external incentives include money and points or “payoff” in a game. For the remainder of this paper we define incentive *I* as a value that is proportional to payoff *V* defined in section Mixed-Motive Games. The key aspect of incentive-based motivation to be embedded in the game theoretic framework in this paper is that different individuals have different intrinsic preferences for incentives. These different intrinsic motivations cause individuals to perceive the payoff matrix specified by a game differently and act according to their own transformation of that matrix.

The following sub-sections describe three incentive-based models of motivation and the different motivational preferences they inspire. While we do not explicitly embed these models in our proposed game theoretic framework, they inform the cases of optimally motivating incentive and corresponding game transformations that we study in section Materials and Methods. The three motives considered are the “influential trio” proposed by Heckhausen and Heckhausen (2008): achievement, affiliation, and power motivation. These theories are the basis of competence-seeking behavior, relationship-building and resource-controlling behavior in humans.

#### Achievement motivation

Achievement motivation drives humans to strive for excellence by improving on personal and societal standards of performance. Perhaps the foremost psychological model of achievement motivation is Atkinson's Risk-Taking Model (RTM) (Atkinson, 1957). It defines achievement motivation in terms of conflicting desires to approach success or avoid failure. Six variables are used: incentive for success (equated with value of success); probability of success (equated with difficulty); strength of motivation to approach success; incentive for avoiding failure; probability of failure; and strength of motivation to avoid failure. Success motivated individuals perceive an inverse linear relationship between incentive and probability of success (Atkinson and Litwin, 1960; Atkinson and Raynor, 1974). They tend to favor goals or actions with moderate incentives which can be interpreted as indicating a moderate probability of success or moderate difficulty. We examine the case of success-motivated individuals in this paper, by examining the case where individuals with a moderate optimally motivating incentive engage in strategic interactions.

#### Affiliation motivation

Affiliation refers to a class of social interactions that seek contact with formerly unknown or little known individuals and maintain contact with those individuals in a manner that both parties experience as satisfying, stimulating and enriching (Heckhausen and Heckhausen, 2008). The need for affiliation is activated when an individual comes into contact with another unknown or little known individual. While theories of affiliation have not been developed mathematically to the extent of the RTM, affiliation can be considered from the perspective of incentive and probability of success (Heckhausen and Heckhausen, 2008). In contrast to success-motivated individuals, individuals high in affiliation motivation may select goals with a higher probability of success and/or lower incentive. This often counter-intuitive preference can be understood as avoiding public competition and conflict. Affiliation motivation is thus an important balance to power motivation, but can also lead to individuals with high affiliation motivation underperforming their achievement motivated colleagues.

#### Power motivation

Power can be described as a domain-specific relationship between two individuals, characterized by the asymmetric distribution of social competence, access to resources or social status (Heckhausen and Heckhausen, 2008). Power is manifested by unilateral behavioral control and can occur in a number of different ways. Types of power include reward power, coercive power, legitimate power, referent power, expert power, and informational power. As with affiliation, power motivation can be considered with respect to incentive and probability of success. Specifically, there is evidence to indicate that the strength of satisfaction of the power motive depends solely on incentive and is unaffected by the probability of success (McClelland and Watson, 1973). Power motivated individuals select high-incentive goals, as achieving these goals gives them significant control of the resources and reinforcers of others.

#### Computational models of achievement, affiliation, and power motivation

**Figure 3:**
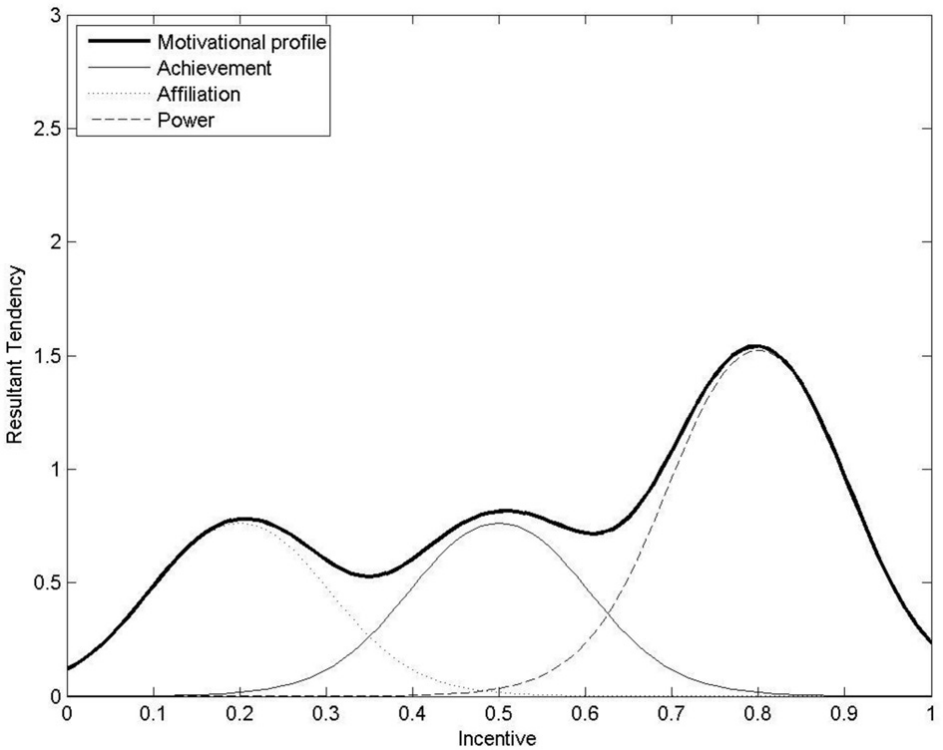
**A computational motive-profile as the sum of achievement, affiliation and power motivation**. The resultant tendency for action is highest for incentive of 0.8 (the optimally motivating incentive for this agent). This agent may be qualitatively classified as “power-motivated” as its optimally motivating incentive is relatively high on the [0, 1] scale for incentive. Image from (Merrick and Shafi, 2011).

Previous work has modeled incentive-based motivation functions computationally for agents with power, achievement, and affiliation motive profiles making one-off decisions (Merrick and Shafi, 2011). For example, Figure 3 shows a possible computational motive profile as a sum of three curves for achievement, affiliation, and power motivation. Unlike utility functions, motivation functions may be non-monotonic and non-concave. The highest peak indicates the level of incentive *I* that produces the strongest resultant motivational tendency *m*(*I*) for action. Assuming a [0, 1] scale for incentive, agents are qualitatively classified as power, achievement or affiliation motivated if their optimally motivating incentive is high, moderate or low, respectively.

## Materials and methods

The previous section establishes that individuals can view incentives differently. Broadly speaking, individuals with strong power, achievement, or affiliation may favor high, moderate, and low incentives, respectively. In a game theoretic setting this suggests that individuals may not play an explicitly described game, but rather act in response to their own idiosyncratic payoff matrix. This phenomenon is not captured by classical game theory or utility based models because of the non-monotonic and non-concave nature of motivation functions.

Our approach in this paper brings the idea of a non-monotonic intrinsic motivation function to game theory by modeling players as having different “optimally motivating incentives.” Optimally motivating incentives are scalar values that represent different motive profiles in a compressed form. Formally, suppose we have two agents *A*_1_ and *A*_2_ playing a mixed-motive game **G**. We denote the optimally motivating incentive of *A*_1_ as I^*^_1_ and the optimally motivating incentive of *A*_2_ as I^*^_2_. I^*^_j_ is thus the value that maximizes the motivation function *m*_*j*_(*I*) of agent *A*_*j*_. This paper is not concerned further with the definition of the function *m*. We focus instead on the game transformations that result from introducing I^*^_j_.

As we have seen, in a two-by-two game, there are four possible outcomes: (*C*, *C*), (*D*, *D*), (*C*, *D*), and (*D*, *C*). The incentive values for each possible outcome from the perspective of the player playing the first listed action are *I* = *R*, *I* = *P*, *I* = *S*, or *I* = *T*. (See section Mixed-Motive Games and Matrix 1.) Suppose each agent *A*_*j*_ wishes to adopt a strategy that results in an outcome that minimizes the difference between *I* and their individual optimally motivating incentive I^*^_j_. That is, each agent wishes to minimize |*I* − I^*^_j_|. This means that agents with different values of I^*^_j_ will perceive the incentives *T*, *S*, *R*, and *P* differently.

We define perceived incentive *I*′_*j*_ as a measure of the perceived value of a particular incentive *I*, for a particular agent *A*_*j*_. If we further suppose that the maximum perceived incentive must be equal to the maximum incentive *I*_*max*_ in the original game, then we can formalize the notion of perceived incentive *I*′_*j*_ as:

$$I_{j}^{\prime }=I_{\max}-|I-I_{j}^{*}|$$

That is, perceived incentive is equal to maximum incentive minus the error between actual and optimal incentive. This means that *I*_max_ only has the highest perceived value if it is closest to the agent's optimally motivating incentive I^*^_j_. In practice the implications are that each incentive *I* will be perceived differently by agents with different optimally motivating incentives I^*^_j_. In addition, the highest actual incentive may not be the highest perceived incentive for all agents.

We can now define the perceived incentives *T*′, *P*′, *S*′, and *R*′ of each incentive in the original game. In PD, Leader, and Chicken the maximum incentive is *I*_max_ = *T* so we have:

$$\begin{array}{l} T_{j}^{\prime }=T-|T-I_{j}^{*}|\;R_{j}^{\prime }=T-|R-I_{j}^{*}|\\ P_{j}^{\prime }=T-|P-I_{j}^{*}|\;S_{j}^{\prime }=T-|S-I_{j}^{*}| \end{array}$$

This gives us the perceived game **G**′ in Matrix 2. For BoS the maximum incentive is *I*_max_ = *S* giving:

$$\begin{array}{l} S_{j}^{\prime }=S-|S-I_{j}^{*}|\;T_{j}^{\prime }=S-|T-I_{j}^{*}|\\ R_{j}^{\prime }=S-|R-I_{j}^{*}|\;P_{j}^{\prime }=S-|P-I_{j}^{*}| \end{array}$$

This produces the perceived game **G**′ in Matrix 3. The next sections examine these perceived games when different values of I^*^_j_ are assumed. We show that the games transform further into a series of new games with different NE depending on the value of I^*^_j_. There are numerous possible transformations of the game, but the remainder of this section focuses in theory on three cases of interest corresponding to individuals with strong power, achievement, and affiliation motivation. The simulations in section Results consider the intermediate cases as well.

Matrix 2. Perceived game **G**′ for PD, Leader, and Chicken.

**Table:** 

	Agent *A*_2_		
		*D*	*C*
Agent *A*_1_	*D*	*T* − |*P* − I^*^_1_|, *T* − |*P* − I^*^_2_|	*T* − |*T* − I^*^_1_|, *T* − |*S* − I^*^_2_|
	*C*	*T* − |*S* − I^*^_1_|, *T* − |*T* − I^*^_2_|	*T* − |*R* − I^*^_1_|,*T* − |*R* − I^*^_2_|

Matrix 3. Perceived game **G**′ for Battle of the Sexes.

**Table:** 

	Agent *A*_2_		
Agent *A*_1_		*D*	*C*
	*D*	*S* − |*P* − I^*^_1_|, *S* − |*P* − I^*^_2_|	*S* − |*T* − I^*^_1_|, *S* − |*S* − I^*^_2_|
	*C*	*S* − |*S* − I^*^_1_|, *S* − |*T* − I^*^_2_|	*S* − |*R* − I^*^_1_|, *S* − |*R* − I^*^_2_|

### Transforming prisoner's dilemma

Using the PD game as an example, we can now consider how a game is transformed into new games, depending on the value of I^*^_j_. Three cases are considered corresponding to individuals with strong power, achievement, and affiliation motivation.

**Case 1 (Power):** The first case examines a range of high optimally motivating incentives: *T* > I^*^_j_ > ½(*T* + *R*). We consider this range “high” because I^*^_j_ is closest to the maximum incentive *T*. This gives us the following transformation of the PD game using Matrix 2 and simplifying the absolute values using the assumption that *T* > I^*^_j_ > ½(*T* + *R*) > *R* > *P* > *S*:

(3) $$T_{j}^{\prime }=T-(T-I_{j}^{*})=I_{j}^{*}$$

(4) $$R_{j}^{\prime }=T-(I_{j}^{*}-R)=T+R-I_{j}^{*}$$

(5) $$P_{j}^{\prime }=T-(I_{j}^{*}-P)=T+P-I_{j}^{*}$$

(6) $$S_{j}^{\prime }=T-(I_{j}^{*}-S)=T+S-I_{j}^{*}$$

*Theorem 1.* For a PD game **G** with *T* > *R* > *P* > *S*, when *T* > I^*^_j_ > ½(*T* + *R*) the perceived game **G**′ is still a valid PD with *T*′_*j*_ > *R*′_*j*_ > *P*′_*j*_ > *S*′_*j*_.

*Proof.* If we assume *R*′_*j*_ ≥ *T*′_*j*_ then we have *T* + *R* − I^*^_j_ ≥ I^*^_j_ which simplifies to ½(*T* + *R*) ≥ I^*^_j_. This contradicts the assumption that *T* > I^*^_j_ > ½(*T* + *R*) so it must be true that *T*′_*j*_ > *R*′_*j*_. If we assume that *P*′_*j*_ ≥ *R*′_*j*_ then we have *T* + *P* − I^*^_j_ ≥ *T* + *R* − I^*^_j_ or *P* ≥ *R* which contradicts the definition of PD. Thus, it must be true that *R*′_*j*_ > *P*′_*j*_. Likewise, if we assume that *S*′_*j*_ ≥ *P*′_*j*_ then we have *T* + *S* − I^*^_j_ ≥ *T* + *P* − I^*^_j_ which simplifies to *S* ≥ *P* which contradicts the definition of PD. Thus, it must be true that *P*′_*j*_ > *S*′_*j*_              □

**Case 2 (Achievement):** The second case examines a range of moderate optimally motivating incentives: ½(*T* + *R*) > I^*^_j_ > *R*. In other words, in this case I^*^_j_ is closest to *R*. This gives us the same basic transformation of the PD game as in Case 1 (Equations 3–6), but now defines a different set of perceived game as follows:

*Theorem 2.* For a PD game *G* with *T* > *R* > *P* > *S*, when ½(*T* + *R*) > I^*^_j_ > *R* the perceived game **G**′ has *R*′_*j*_ > *T*′_*j*_ and *P*′_*j*_ > *S*′_*j*_.

*Proof.* If we assume *T*′_*j*_ ≥ *R*′_*j*_ then we have I^*^_j_ ≥ *T* + *R* − I^*^_j_ which simplifies to I^*^_j_ ≥ ½(*T* + *R*). This contradicts the assumption in this case that ½(*T* + *R*) > I^*^_j_ so it must be true that *R*′_*j*_ > *T*′_*j*_. If we assume that *S*′_*j*_ ≥ *P*′_*j*_ then we have *T* + *S* − I^*^_j_ ≥ *T* + *P* − I^*^_j_ which simplifies to *S* ≥ *P* which contradicts the definition of PD. Thus, it must be true that *P*′_*j*_ > *S*′_*j*_              □

**Case 3 (Affiliation):** The third case examines a range of low optimally motivating incentives: ½(*P* + *S*) > I^*^_j_ > *S*. We consider this range “low” because I^*^_j_ is closest to *S*. This gives us the following transformation of the PD game using Matrix 2 and simplifying absolute values:

$$\begin{array}{l} T_{j}^{\prime }=T-(T-I_{j}^{*})=I_{j}^{*}\\ R_{j}^{\prime }=T-(R-I_{j}^{*})=T+I_{j}^{*}-R\\ P_{j}^{\prime }=T-(P-I_{j}^{*})=T+I_{j}^{*}-P\\ S_{j}^{\prime }=T-(I_{j}^{*}-S)=T+S-I_{j}^{*} \end{array}$$

*Theorem 3.* For a PD game **G** with *T* > *R* > *P* > *S*, when ½(*P* + *S*) > I^*^_j_ > *S* the perceived game **G**′ has *S*′_*j*_ > *P*′_*j*_ > *R*′_*j*_ > *T*′_*j*_.

*Proof.* If we assume *P*′_*j*_ = *S*′_*j*_ then we have *T* + I^*^_j_ − *P* ≥ *T* + *S* − I^*^_j_ which simplifies to I^*^_j_ ≥ ½(*P* + *S*). This contradicts the assumption that ½(*P* + *S*) > I^*^_j_. Thus, it must be true that *S*′_*j*_ > *P*′_*j*_. If we assume *R*′_*j*_ ≥ *P*′_*j*_ then we have *T* + I^*^_j_ − *R* ≥ *T* + I^*^_j_ − *P* which simplifies to *P* ≥ *R*. This contradicts the definition of PD. Thus, it must be true that *P*′_*j*_ > *R*′_*j*_. Likewise, if we assume *T*′_*j*_ ≥ *R*′_*j*_ then we have I^*^_j_ ≥ *T* + I^*^_j_ − *R* which simplifies to *R* ≥ *T*. This contradicts the definition of PD. Thus, it must be true that *R*′_*j*_ > *T*′_*j*_              □

**Figure 4:**
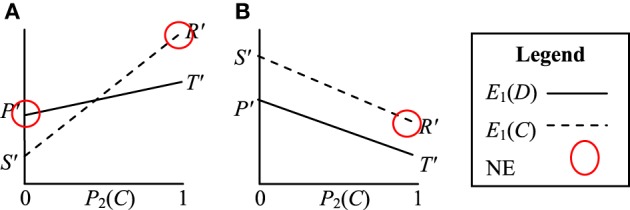
Visualization of the Prisoner's Dilemma game when perceived by agents with optimally motivating incentives of (A) ½(*T* + *R*) > I^*^_j_ > *R* and (B) ½(*P* + *S*) > I^*^_L_ > *S*. The pure strategy Nash Equilibria (NE) are circled.

The three cases above result in a number of different perceived games. Case 1 still results in a valid PD game, but in Case 2 and Case 3 the perceived games are new games. An example of the payoff structure of the new perceived game from Case 2 is visualized in Figure 4A. In this game *E*_1_(*D*) > *E*_1_(*C*) for *P*_2_(*C*) = 0 and *E*_1_(*D*) < *E*_1_(*C*) for *P*_2_(*C*) = 1. *E*_1_(*D*) and *E*_1_(*C*) intersect at:

$$P_{2}(C)=\frac{P^{\prime }-S^{\prime }}{R^{\prime }-S^{\prime }-T^{\prime }+P^{\prime }}=M$$

There are now two pure NE and the strategy that emerges depends on the initial values of *P*_1_(*C*) and *P*_2_(*C*). If *P*_1_(*C*) + *P*_2_(*C*) > 2*M* at *t* = 0 then the (*C*, *C*) equilibrium will emerge. Alternatively if *P*_1_(*C*) + *P*_2_(*C*) < 2*M* at *t* = 0 then the (*D*, *D*) equilibrium will emerge.

In Case 3 the agents also do not perceive a PD game. The perceived game in this case is visualized in Figure 4B. In this game *E*_1_(*C*) > *E*_1_(*D*) for all *P*_2_(*C*). The (*C*, *C*) strategy is now dominant, indicating that the agents will tend to evolve cooperative (*C*, *C*) strategies over time.

### Transforming leader

We can follow the same process to construct perceived versions of Leader.

**Case 1 (Power):** The first case again examines a range of high optimally motivating incentives: *T* > I^*^_j_ > ½(*T* + *S*). This gives us the same basic transformations in Equations 3–6, and the perceived game is still a Leader game.

*Theorem 1.* In a Leader game **G** with *T* > *S* > *R* > *P*, when *T* > I^*^_j_ > ½(*T* + *S*) the perceived game **G′** is still a valid Leader game *T*′_*j*_ > *S*′_*j*_ > *R*′_*j*_ > *P*′_*j*_.

*Proof.* If we assume *S*′_*j*_ ≥ *T*′_*j*_ then we have *T* + *S* − I^*^_j_ ≥ I^*^_j_ which simplifies to ½(*T* + *S*) ≥ I^*^_j_. This contradicts the assumption in this case that *T* > I^*^_j_ > ½(*T* + *S*) so it must be true that *T*′_*j*_ > *S*′_*j*_. If we assume that *R*′_*j*_ ≥ *S*′_*j*_ then we have *T* + *R* − I^*^_j_ ≥ *T* + *S* − I^*^_j_ which simplifies to *R* ≥ *S* which contradicts the definition of Leader. Thus, it must be true that *S*′_*j*_ > *R*′_*j*_. Likewise, if we assume that *P*′_*j*_ ≥ *R*′_*j*_ then we have *T* + *P* − I^*^_j_ ≥ *T* + *R* − I^*^_j_ which simplifies to *P* ≥ *R* which contradicts the definition of Leader. Thus, it must be true that *R*′_*j*_ > *P*′_*j*_              □

**Case 2 (Achievement):** The second case examines a range of moderate-high optimally motivating incentive: ½(*T* + *S*) > I^*^_j_ > *S*. This also gives us the transformations in Equations 3–6, but the perceived game is no longer a Leader game. In fact, a number of interesting variations occur:

*Lemma 1.* In a Leader game **G** with *T* > *S* > *R* > *P*, when ½(*T* + *S*) > I^*^_j_ > *S* the perceived game **G′** has *S*′_*j*_ > *T*′_*j*_ and *R*′_*j*_ > *P*′_*j*_.

*Proof.* If we assume *T*′_*j*_ ≥ *S*′_*j*_ then we have I^*^_j_ ≥ *T* + *S* − I^*^_j_ which simplifies to I^*^_j_ ≥ ½(*T* + *S*). This contradicts the assumption in this case that ½(*T* + *S*) > I^*^_j_ so it must be true that *S*′_*j*_ > *T*′_*j*_. If we assume that *P*′_*j*_ ≥ *R*′_*j*_ then we have *T* + *P* − I^*^_j_ ≥ *T* + *R* − I^*^_j_ which simplifies to *P* ≥ *R* which contradicts the definition of Leader. Thus, it must be true that *R*′_*j*_ > *P*′_*j*_              □

*Theorem 2.* In a Leader game **G** with *T* > *S* > *R* > *P*, when ½(*T* + *S*) > I^*^_j_ > *S* and I^*^_j_ > ½(*T* + *R*) the perceived game **G**′ is a BoS game *S*′_*j*_ > *T*′_*j*_ > *R*′_*j*_ > *P*′_*j*_

*Proof. S*′_*j*_ > *T*′_*j*_ and *R*′_*j*_ > *P*′_*j*_ by Lemma 3.2.2. I^*^_j_ > ½(*T* + *R*) expands to I^*^_j_ > *T* + *R* − I^*^_j_. Substitution of Equations 3–4 gives us *T*′_*j*_ > *R*′_*j*_              □

*Theorem 3.* In a Leader game **G** with *T* > *S* > *R* > *P*, when ½(*T* + *S*) > I^*^_j_ > *S* and I^*^_j_ < ½(*T* + *R*) the perceived game **G**′ is *S*′_*j*_ > *R*′_*j*_ > *T*′_*j*_ > *P*′_*j*_.

*Proof. S*′_*j*_ > *T*′_*j*_ and *R*′_*j*_ > *P*′_*j*_ by Lemma 3.2.2. I^*^_j_ < ½(*T* + *R*) expands to I^*^_j_ < *T* + *R* − I^*^_j_. Substitution of Equations 3–4 gives us *T*′_*j*_ < *R*′_*j*_.              □

**Case 3 (Affiliation):** The third case examines a range of low optimally motivating incentives: ½(*R* + *P*) > I^*^_j_ > *P*. This gives us the following transformation:

(7) $$T_{j}^{\prime }=T-[T-I_{j}^{*}]=I_{j}^{*}$$

(8) $$R_{j}^{\prime }=T-[R-I_{j}^{*}]=T+I_{j}^{*}-R$$

(9) $$P_{j}^{\prime }=T-[I_{j}^{*}-P]=T+P-I_{j}^{*}$$

(10) $$S_{j}^{\prime }=T-[S-I_{j}^{*}]=T+I_{j}^{*}-S$$

*Theorem 4.* In a Leader game **G** with *T* > *S* > *R* > *P*, when ½(*R* + *P*) > I^*^_j_ > *P* the perceived game **G**′ is *P*′_*j*_ > *R*′_*j*_ > *S*′_*j*_ > *T*′_*j*_.

*Proof.* If we assume *R*′_*j*_ ≥ *P*′_*j*_ we have *T* + I^*^_j_ − *R* ≥ *T* + *P* − I^*^_j_ which simplifies to I^*^_j_ ≥ 1/2(*R* + *P*) which contradicts the assumption that 1/2(*R* + *P*) > I^*^_j_. If we assume *S*′_*j*_ ≥ *R*′_*j*_ we have *T* + I^*^_j_ − *S* ≥ *T* + I^*^_j_ − *R* or *R* ≥ *S* which contradicts the definition of Leader. Thus, it must be true that *R*′_*j*_ > *S*′_*j*_. Likewise if we assume *T*′_*j*_ ≥ *S*′_*j*_ we have I^*^_j_ ≥ *T* + I^*^_j_ − *S* or *S* ≥ *T* which contradicts the definition of Leader. Thus, it must be true that *S*′_*j*_ > *T*′_*j*_              □

### Transforming chicken

We can follow the same process again to construct the perceived versions of Chicken. Proofs are omitted for brevity.

**Case 1 (Power):** The first case again assumes a high optimally motivating incentive: *T* > I^*^_j_ > 1/2(*T* + *R*). This gives us the transformation in Equations 3–6, and the perceived game is a Chicken game:

*Theorem 1.* For a Chicken game **G** with *T* > *R* > *S* > *P*, when *T* > I^*^_j_ > 1/2(*T* + *R*) the perceived game **G**′ is still a valid Chicken game *T*′_*j*_ > *R*′_*j*_ > *S*′_*j*_ > *P*′_*j*_.

*Proof.* Omitted.              □

**Case 2 (Achievement):** The second case again assumes a moderate-high optimally motivating incentive: ½(*T* + *R*) > I^*^_j_ > *R*. This also gives us the transformation in Equations 3–6, but the perceived game is no longer a Chicken game:

*Theorem 2.* For a Chicken game **G** with *T* > *R* > *S* > *P*, when ½(*T* + *R*) > I^*^_j_ > *R* the perceived game **G**′ has *R*′_*j*_ > *T*′_*j*_ and *S*′_*j*_ > *P*′_*j*_.

*Proof.* Omitted.              □

**Case 3 (Affiliation):** The third case again assumes a low optimally motivating incentive: ½(*S* + *P*) > I^*^_j_ > *P*. This gives us the transformations in Equations 7–10.

*Theorem 3.* For a Chicken game **G** with *T* > *R* > *S* > *P*, when ½(*S* + *P*) > I^*^_j_ > *P* the perceived game **G**′ is *P*′_*j*_ > *S*′_*j*_ > *R*′_*j*_ > *T*′_*j*_

*Proof.* Omitted.              □

### Transforming battle of the sexes

Finally, we can follow the process above to construct the perceived versions of BoS.

**Case 1 (Power):** The first case again assumes a high optimally motivating incentive: *S* > I^*^_j_ > ½(*T* + *S*). This gives us the following transformation of the BoS game:

(11) $$T_{j}^{\prime }=S-(I_{j}^{*}-T)=S+T-I_{j}^{*}$$

(12) $$R_{j}^{\prime }=S-(I_{j}^{*}-R)=S+R-I_{j}^{*}$$

(13) $$P_{j}^{\prime }=S-(I_{j}^{*}-P)=S+P-I_{j}^{*}$$

(14) $$S_{j}^{\prime }=S-(S-I_{j}^{*})=I_{j}^{*}$$

*Theorem 1.* For a BoS game **G** with *S* > *T* > *R* > *P*, when *S* > I^*^_j_ > ½(*T* + *S*) the perceived game **G**′ is still a valid BoS game *S*′_*j*_ > *T*′_*j*_ > *R*′_*j*_ > *P*′_*j*_.

*Proof.* Omitted.              □

**Case 2 (Achievement):** The second case again assumes a moderate-high optimally motivating incentive: ¡(*T* + *S*) > I^*^_j_ > *T*. This gives us the transformation of the BoS game in Equations 11–14, but the perceived game is no longer a BoS.

*Lemma 1.* For a BoS game **G** with *S* > *T* > *R* > *P*, when ½(*T* + *S*) > I^*^_j_ > *T* the perceived game **G**′ has *T*′_*j*_ > *S*′_*j*_ and *R*′_*j*_ > *P*′_*j*_.

*Proof.* If we assume *S*′_*j*_ ≥ *T*′_*j*_ then we have I^*^_j_ ≥ *S* + *T* − I^*^_j_ which simplifies to I^*^_j_ ≥ ½(*T* + *S*) which contradicts the assumption that ½(*T* + *S*) > I^*^_j_. Thus, it must be true that *S*′_*j*_ > *T*′_*j*_. If we assume *P*′_*j*_ ≥ *R*′_*j*_ then we have *S* + *P* − I^*^_j_ ≥ *S* + *R* − I^*^_j_ which simplifies to *P* ≥ *R* which contradicts the definition of BoS. Thus, it must be true that *R*′_*j*_ > *P*′_*j*_              □

*Theorem 2.* For a BoS game **G** with *S* > *T* > *R* > *P*, when ½(*T* + *S*) > I^*^_j_ > *T* and I^*^_j_ > ½(*S* + *R*) the perceived game **G**′ is a Leader game *T*′_*j*_ > *S*′_*j*_ > *R*′_*j*_ > *P*′_*j*_.

*Proof. T*′_*j*_ > *S*′_*j*_ and *R*′_*j*_ > *P*′_*j*_ by Lemma 3.4.2. I^*^_j_ > ½(*S* + *R*) expands to I^*^_j_ > *S* + *R* − I^*^_j_. Substitution of Equations 14 and 12 gives us *S*′_*j*_ > *R*′_*j*_              □

*Theorem 3.* For a BoS game **G** with *S* > *T* > *R* > *P*, when ½(*T* + *S*) > I^*^_j_ > *T* and I^*^_j_ < ½(*S* + *R*) the perceived game **G**′ is a Chicken game *T*′_*j*_ > *R*′_*j*_ > *S*′_*j*_ > *P*′_*j*_.

*Proof. T*′_*j*_ > *S*′_*j*_ and *R*′_*j*_ > *P*′_*j*_ by Lemma 3.4.2. I^*^_j_ < ½(*S* + *R*) expands to I^*^_j_ < *S* + *R* − I^*^_j_. Substitution of Equations 14 and 12 gives us *S*′_*j*_ < *R*′_*j*_              □

**Case 3 (Affiliation):** The third case again assumes a low optimally motivating incentive: ½(*R* + *P*) > I^*^_j_ > *P*. This gives us the following transformation of the BoS game:

$$\begin{array}{l} T_{j}^{\prime }=S-(T-I_{j}^{*})=S+I_{j}^{*}-T\\ R_{j}^{\prime }=S-(R-I_{j}^{*})=S+I_{j}^{*}-R\\ P_{j}^{\prime }=S-(I_{j}^{*}-P)=S+P-I_{j}^{*}\\ S_{j}^{\prime }=S-(S-I_{j}^{*})=I_{j}^{*} \end{array}$$

*Theorem 4.* For a BoS game **G** with *S* > *T* > *R* > *P*, when ½(*R* + *P*) > I^*^_j_ > *P* the perceived game **G**′ is *P*′_*j*_ > *R*′_*j*_ > *T*′_*j*_ > *S*′_*j*_.

*Proof.* If we assume *R*′_*j*_ ≥ *P*′_*j*_ then we have *S* + I^*^_j_ − *R* ≥ *S* + *P* − I^*^_j_ or I^*^_j_ ≥ ½(*R* + *P*) which contradicts the assumption that ½(*R* + *P*) > I^*^_j_. Thus, it must be true that *P*′_*j*_ > *R*′_*j*_. If we assume that *T*′_*j*_ ≥ *R*′_*j*_ then we have *S* + I^*^_j_ − *T* ≥ *S* + I^*^_j_ − *R* or *R* ≥ *T* which contradicts the definition of BoS. Thus, it must be true that *R*′_*j*_ > *T*′_*j*_. Likewise, if we assume that *S*′_*j*_ ≥ *T*′_*j*_ then we have I^*^_j_ ≥ *S* + I^*^_j_ − *T* or *T* ≥ *S* which contradicts the definition of BoS. Thus, it must be true that *T*′_*j*_ > *S*′_*j*_              □

## Results

This section presents simulations of the each of the four games studied in section Materials and Methods played by agents with optimally motivating incentives conforming to the three cases studied, as well as the intermediate cases not studied above. We use two-population replicator dynamics to model cultural learning when mixed-motive games are played iteratively. We demonstrate that individuals with different optimally motivating incentives may adopt different strategies when playing a particular game, or may learn at different rates. We also discuss how the NE of the transformed games reflects a number of results from human experiments that are not well-modeled by the NE of the original game.

### Prisoners' dilemma

**Figure 5:**
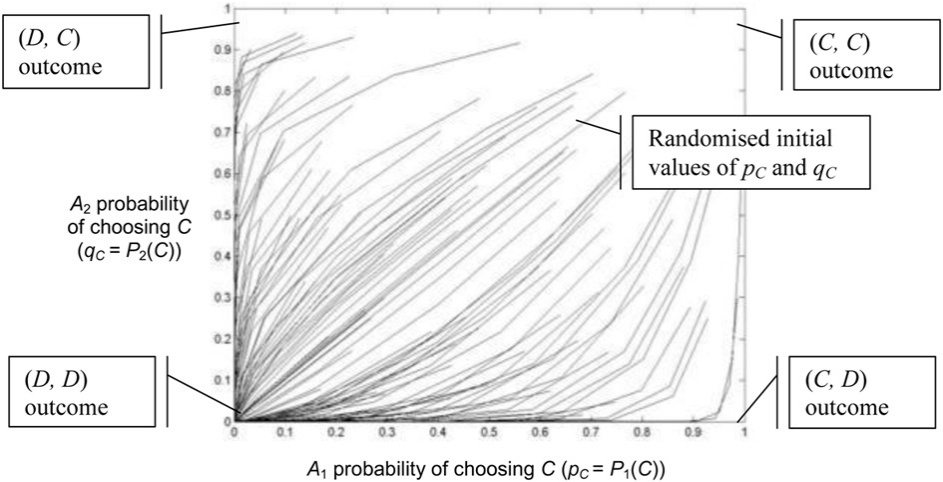
**Simulation of one hundred pairs of agents playing thirty iterations of the Prisoner's Dilemma game**. All agents have I^*^_j_ = 4.0, but initial values of *p*_*C*_ and *q*_*C*_ are randomized.

**Figure 6:**
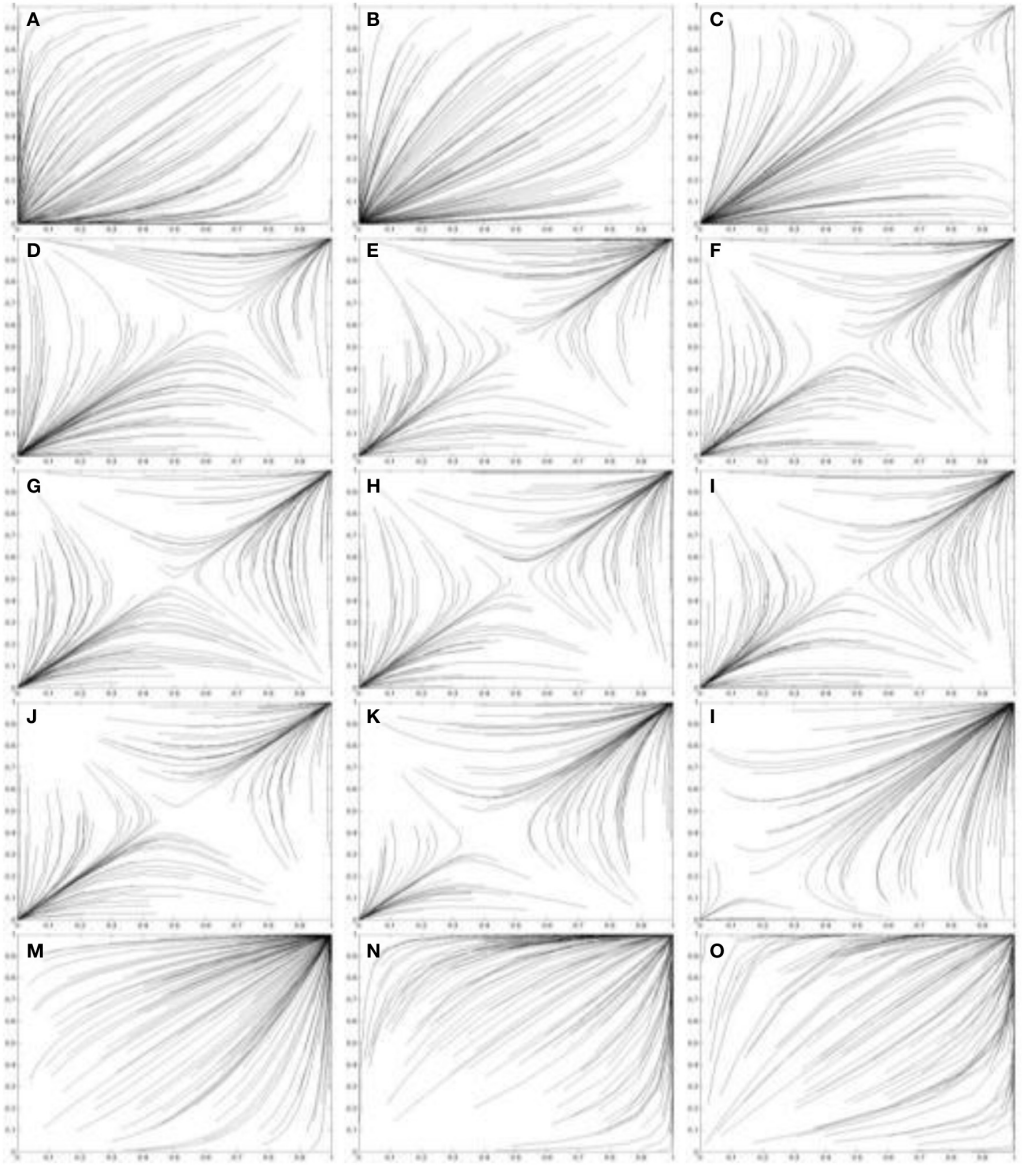
**Simulations of one hundred pairs of agents playing thirty iterations of the Prisoner's Dilemma game**. Agents share different values of I^*^_j_ in each simulation. **(A)** I^*^_j_ = 3.8; **(B)** I^*^_j_ = 3.6; **(C)** I^*^_j_ = 3.4; **(D)** I^*^_j_ = 3.2; **(E)** I^*^_j_ = 3.0; **(F)** I^*^_j_ = 2.8; **(G)** I^*^_j_ = 2.6; **(H)** I^*^_j_ = 2.4; (i) I^*^_j_ = 2.2; **(J)** I^*^_j_ = 2.0; **(K)** I^*^_j_ = 1.8; **(L)** I^*^_j_ = 1.6; **(M)** I^*^_j_ = 1.4; **(N)** I^*^_j_ = 1.2; (o) I^*^_j_ = 1.0. Initial values of *p*_*C*_ and *q*_*C*_ are randomized. See Figure 5 for legend.

Figures 5, 6 use the two population replicator dynamics in Equations 1 and 2 to simulate one hundred pairs of agents (*A*_1_ and *A*_2_) playing the iterated PD (IPD^1^) game:

$$G=\left[\begin{array}{cc} 2 & 4\\ 1 & 3 \end{array}\right]$$

The initial probabilities *p*_*C*_ (for agents *A*_1_) and *q*_*C*_ (for agents *A*_2_) are randomized and the agent pairs learn while playing thirty consecutive games. A range of [1, 4] is assumed for incentive. The lines in Figure 5 trace the learned values of *p*_*C*_ and *q*_*C*_ over time. In Figure 5 all agents have a “high” optimally motivating incentive I^*^_1_ = I^*^_2_ = 4.0, representing power-motivated individuals. We see that the perceived games are identical to the original game, ie: **G**′_1_ = **G**′_2_ = *G* and all agent pairs tend to converge on the (*D*, *D*) equilibrium over time.

In Figure 6 the agents share progressively lower values of I^*^_1_ and I^*^_2_, ranging from I^*^_1_ = I^*^_2_ = 3.8 in Figure 6A to I^*^_1_ = I^*^_2_ = 1.0 in Figure 6O. Figures 6A,B show Case 1 games in which the (*D*, *D*) outcome emerges as the equilibrium as predicted by Theorem 2.1.1. These agents still perceive a PD game. In contrast, Figures 6C,D show Case 2 games in which some agents converge on the (*C*, *C*) equilibrium and some on the (*D*, *D*) equilibrium, as predicted by Theorem 2.1.2. The equilibrium approached by the agent pairs in this case depends on their initial values of *p*_*C*_ and *q*_*C*_. In Figures 6E–L the (*C*, *C*) outcome becomes more frequent as the values of I^*^_1_ and I^*^_2_ decrease. Figures 6M,N shows Case 3 games in which all agents converge on the (*C*, *C*) equilibrium as predicted by Theorem 2.1.3.

In general, these results support the idea proposed by Johnson et al. (2002), that individual variation means that true PD scenarios occur relatively infrequently in nature. Johnson et al. (2002) show that if there is variance in perception of twice the payoff interval in a linear PD game (a game in which the intervals between *T*, *R*, *S*, and *P* are the same) then only 15.8% remain valid PD games. Our transformations show that a true PD scenario will only occur if both agents have optimally motivating incentives that fall in the range *T* > I^*^ > ½(*T* + *R*). If we assume I^*^ can only fall within the range of *T* ≥ I^*^ ≥ *S*, the fraction *v* of valid PD games will be:

$$v=\frac{T-1/2(T+R)}{T-S}=\frac{T-R}{2(T-S)}$$

In a linear PD game 3(*T* − *R*) = (*T* − *S*) so *v* = 1/6 = 16.6% if we assume a uniform distribution of optimally motivating incentives. This is, qualitatively speaking, similar to the result proposed by Johnson et al. (2002), and offers support for our methodology for modeling differences in motivations.

Case 1 and Case 2 also provide computational insight into some of the findings reported by Terhune (1968). Terhune observed pairs of humans classified as either power, affiliation and achievement motivated playing single-shot and iterative PD games in controlled conditions. One of these experiments observed the influence of the first trial outcome on different types of people. He found that if the first outcome was (*C*, *C*), pairs of achievement motivated individuals had the highest subsequent proportion of (*C*, *C*) outcomes (46.8%). In contrast, power motivated individuals had (*C*, *C*) outcomes only 9.4% of the time after a (*C*, *C*) outcome on the first trial. In other words people with different motives respond differently to the same experience (in this case the first trial outcome). The results above suggest that this can be captured computationally using our model by using high values of I^*^ for power motivated individuals, so that they tend to perceive a Case 1 game and lower values of I^*^ for achievement motivated individuals, so that they tend to perceive a Case 2 game. A further discussion of this avenue for future work is made in section Human-Computer Interaction.

The Case 3 result is perhaps less instructive from a human modeling perspective, but is still useful from an artificial systems perspective. If we wish to design agents that will cooperate when faced with PD situations, then we can use agents with low optimally motivating incentives in the range ½(*P* + *S*) > I^*^_1_ > *S*. These agents perceive a game with a dominant (*C*, *C*) strategy and will thus tend to evolve cooperative strategies over time. Likewise, if we wish to model “martyrs” then an agent *A*_1_ with ½(*P* + *S*) > I^*^_1_ > *S* will be a martyr (*C* chooser) when playing an agent *A*_2_ with *T* > I^*^_2_ > ½(*T* + *R*). This type of personality modeling has application to areas such as believable non-player characters (NPCs) in computer games.

### Leader

If we consider Case 1(power-motivated) agents playing the leader game, we see that *E*_1_(*C*) > *E*_1_(*D*) for *P*_2_(*C*) = 0 and *E*_1_(*D*) > *E*_1_(*C*) for *P*_2_(*C*) = 1. *E*_1_(*C*) and *E*_1_(*D*) intersect at the point:

$$P_{2}(C)=\frac{S-P}{2I^{*}+S-P-T-R}$$

Now, suppose we have two pairs of players. The first pair of players have optimally motivating incentives I^*^_1_ = I^*^_2_ = I^*^_j_. The second pair of players have optimally motivating incentives I^*^_1_ = I^*^_2_ = I^*^_k_ such that I^*^_j_ > I^*^_k_. Substitution gives us

$$\frac{S-P}{2I_{j}^{*}+S-P-T-R}<\frac{S-P}{2I_{k}^{*}+S-P-T-R}$$

**Figure 7:**
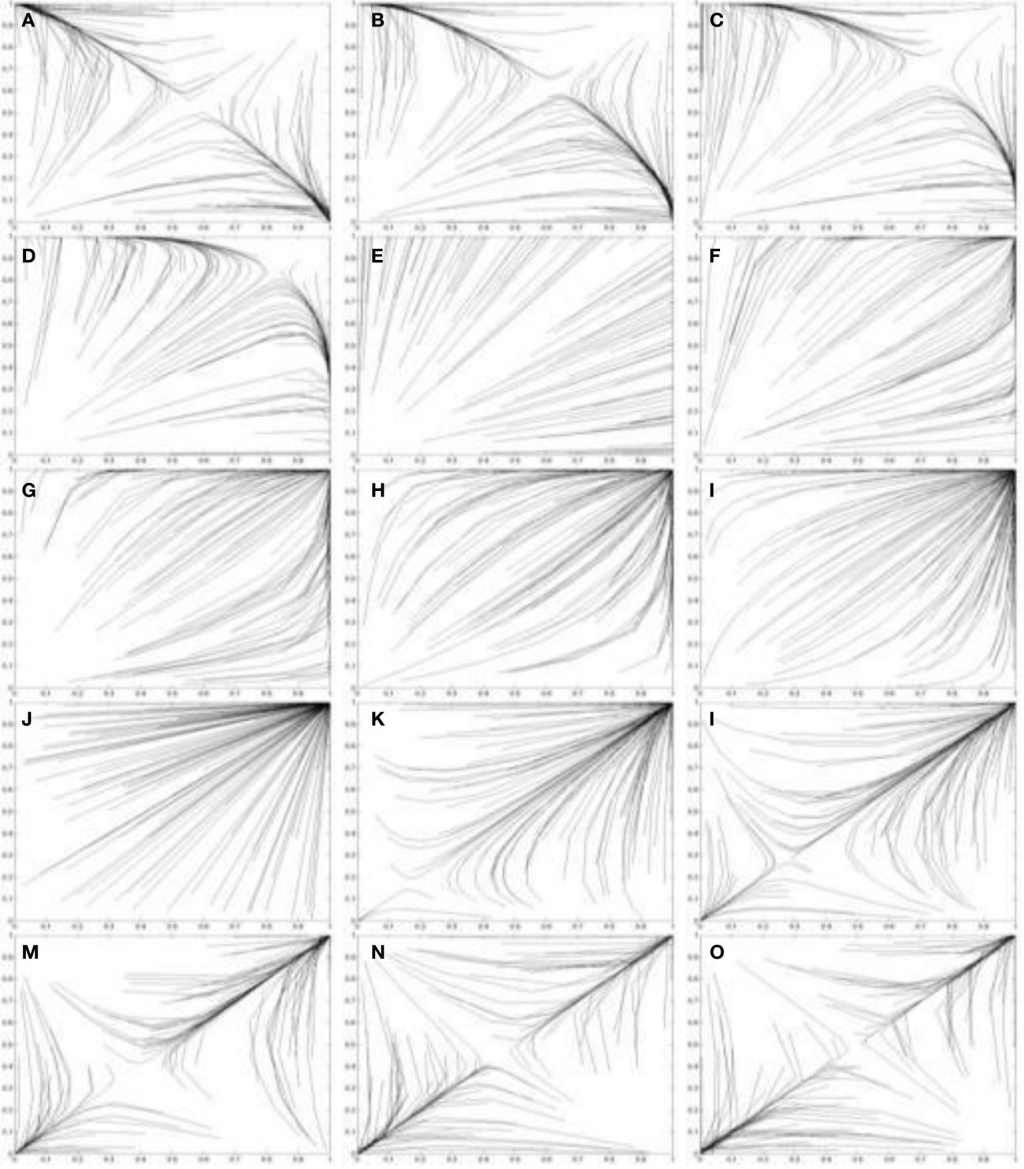
**Simulations of one hundred pairs of agents playing thirty iterations of the Leader game**. Agents share different values of I^*^_j_ in each simulation. **(A)** I^*^_j_ = 3.8; **(B)** I^*^_j_ = 3.6; (c) I^*^_j_ = 3.4; **(D)** I^*^_j_ = 3.2; **(E)** I^*^_j_ = 3.0; **(F)** I^*^_j_ = 2.8; **(G)** I^*^_j_ = 2.6; **(H)** I^*^_j_ = 2.4; **(I)** I^*^_j_ = 2.2; **(J)** I^*^_j_ = 2.0; **(K)** I^*^_j_ = 1.8; **(L)** I^*^_j_ = 1.6; **(M)** I^*^_j_ = 1.4; **(N)** I^*^_j_ = 1.2; **(O)** I^*^_j_ = 1.0. Initial values of *p*_*C*_ and *q*_*C*_ are randomized. See Figure 5 for legend.

That is, *P*_*j*_(*C*) < *P*_*k*_(*C*). In other words the probability of conceding right of way increases in games between players with weaker power motivation, although the equilibria are still at (*C*, *D*) and (*D*, *C*) as indicated by Theorem 2.2.1. This phenomenon is evident in the simulations in Figure 7. Figure 7 uses the two population replicator dynamics in Equations 1 and 2 to simulate one hundred pairs of learning agents (*A*_1_ and *A*_2_) playing the Leader game:

$$G=\left[\begin{array}{cc} 1 & 4\\ 3 & 2 \end{array}\right]$$

The Case 1 simulations are shown in Figures 7A,B and the trend to concede is evident in the progressively less direct paths the agent's take to the equilibria. As I^*^_j_ is further decreased in Case 2 (achievement motivated agents), two types of perceived games occur. Either the game is perceived as a BoS game (Theorem 2.2.3), or as a game with a dominant (*C*, *C*) strategy (Theorem 2.2.4).

The Leader game is perceived as a BoS game when ½(*T* + *S*) > I^*^_j_ > *S* and I^*^_j_ = ½(*T* + *R*). The payoff structure for a BoS game is visualized in Figure 2C. Figures 7C,D simulates the behavior of agents that perceive a Leader game as a BoS game. The paths taken to the (*C*, *D*) and (*D*, *C*) equilibria by these agents are quite indirect as both are initially motivated to concede right of way by their perception of leadership as an act of sacrifice. Leader-follower behavior [(*C*, *D*) or (*D*, *C*)] does emerge, but it does so more slowly than for agents with high values of I^*^_j_ because leadership is now perceived as an act of sacrifice.

Figures 7E–J shows simulations of games between agents with *S* > I^*^_j_ > *R*. These agents perceive games of the form *S*′_*j*_ > *R*′_*j*_ > *T*′_*j*_ > *P*′_*j*_ with dominant (*C*, *C*) strategies. As a result, leadership behavior does not emerge as an equilibrium as the agents always concede right of way. In Case 3(affiliation motivated agents) there are two pure equilibria in the perceived game: (*D*, *D*) and (*C*, *C*). The Case 3 payoff structure is simulated in Figures 7M,N. The emergent equilibrium strategy for any pair of agents depends on the initial values of *P*_1_(*C*) and *P*_2_(*C*). If *P*_1_(*C*) + *P*_2_(*C*) > 2*M* at *t* = 0 then the (*C*, *C*) equilibrium will occur over time. Alternatively if *P*_1_(*C*) + *P*_2_(*C*) < 2*M* at *t* = 0 then the (*D*, *D*) equilibrium will occur over time. These pure strategy equilibria preclude the emergence of leader-follower behavior and result, instead, in collisions (both players driving) or procrastination (both players conceding right of way). Thus, to achieve leaders and followers agents with high values of I^*^ are required.

### Chicken

In the chicken game, Case 1(power-motivated) agents also perceive a valid Chicken game resulting in the emergence of an “exploiter” agent. However, with a small reduction in I^*^_j_ Case 2 (achievement motivated) agents perceive a transformed game in which the more cautious (*C*, *C*) strategy is dominant (Theorem 2.3.2). This is, in fact, the most common perceived game, covering ½(*T* + *R*) > I^*^_j_ > ½(*S* + *P*). This can be thought of as reflecting the real-world reluctance to engage in a game of Chicken, which is in principle the same as playing and choosing *C* (Colman, 1982).

**Figure 8:**
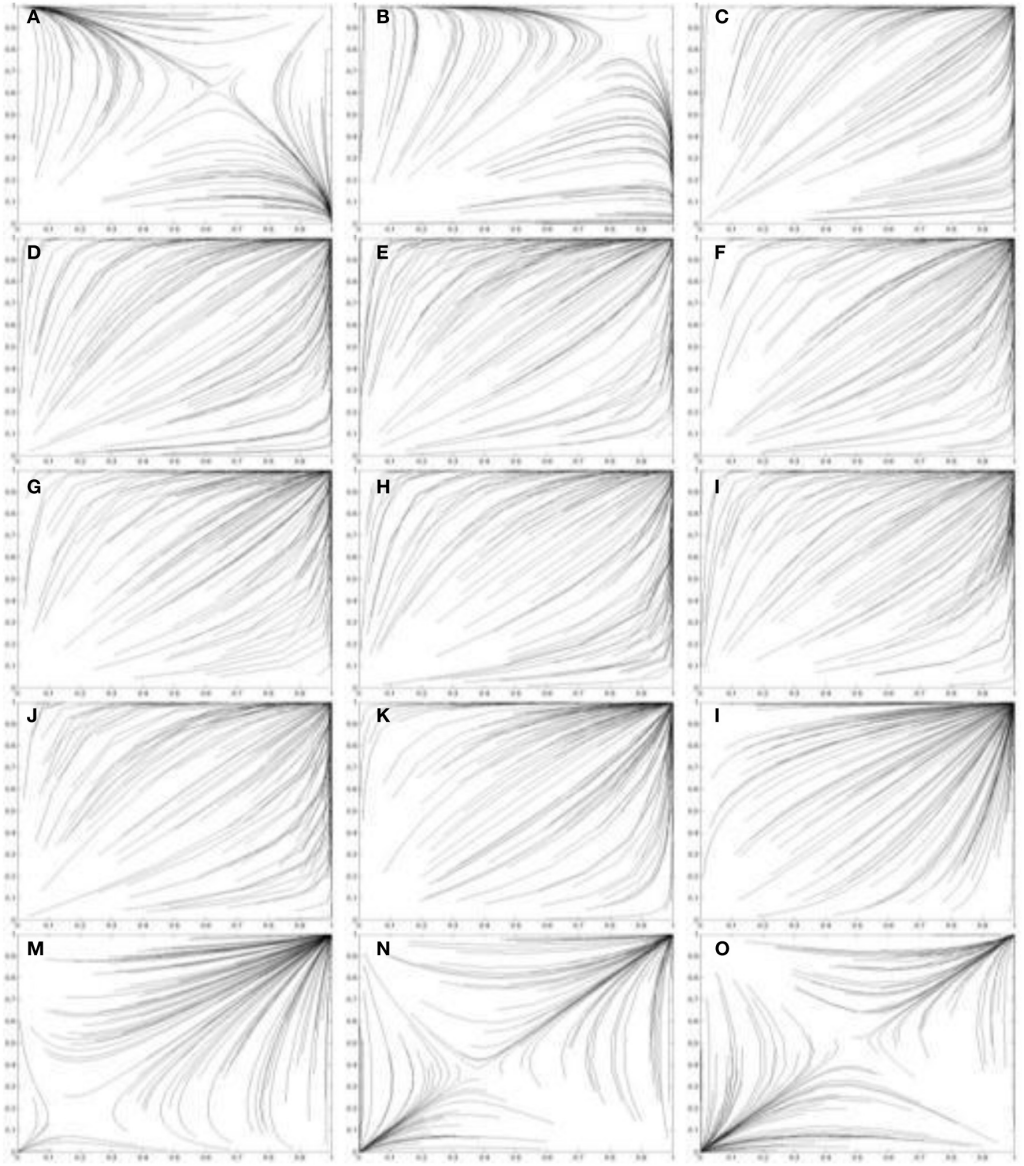
**Simulations of one hundred pairs of agents playing thirty iterations of the Chicken game**. Agents share different values of I^*^_j_ in each simulation. **(A)** I^*^_j_ = 3.8; **(B)** I^*^_j_ = 3.6; **(C)** I^*^_j_ = 3.4; **(D)** I^*^_j_ = 3.2; **(E)** I^*^_j_ = 3.0; **(F)** I^*^_j_ = 2.8; **(G)** I^*^_j_ = 2.6; **(H)** I^*^_j_ = 2.4; **(I)** I^*^_j_ = 2.2; **(J)** I^*^_j_ = 2.0; **(K)** I^*^_j_ = 1.8; **(L)** I^*^_j_ = 1.6; **(M)** I^*^_j_ = 1.4; **(N)** I^*^_j_ = 1.2; **(O)** I^*^_j_ = 1.0. Initial values of *p*_*C*_ and *q*_*C*_ are randomized. See Figure 5 for legend.

The prevalence of the perceived dominant (*C*, *C*) strategy is evidenced in the simulations in Figure 8. Figure 8 uses the two population replicator dynamics in Equations 1 and 2 to simulate one hundred pairs of learning agents (*A*_1_ and *A*_2_) playing the Chicken game:

$$G=\left[\begin{array}{cc} 1 & 4\\ 2 & 3 \end{array}\right]$$

Figures 8C–L all show agents approaching the (*C*, *C*) equilibrium. One other case does exist (Case 3) in which the perceived game has two pure NE: (*D*, *D*) and (*C*, *C*). The emergent equilibrium for two agents depends on the initial values of *P*_1_(*C*) and *P*_2_(*C*). If *P*_1_(*C*) + *P*_2_(*C*) > 2*M* at *t* = 0 then the (*C*, *C*) equilibrium will occur over time. Alternatively if *P*_1_(*C*) + *P*_2_(*C*) < 2*M* at *t* = 0 then the (*D*, *D*) equilibrium will occur over time. These pure strategy equilibria result in either certain collision (both players driving on) or mutually cautious behavior (both players swerving to avoid a collision). Examples of Case 3 agents interacting are shown in Figures 7M,N.

Comparison of Case 1 and Case 3 demonstrates how the same outcome may result from different motives. In Case 1 the (*D*, *D*) outcome results from a preference for high incentives. In Case 3 the (*D*, *D*) outcome results from a preference for low incentives to avoid conflict. The strategy clearly backfires, but this sort of trend has been observed in a general sense in humans. Individuals with high affiliation motivation have been observed to underperform their achievement motivated colleagues precisely because their desire to avoid conflict situations often means they also miss opportunities to cooperate (Heckhausen and Heckhausen, 2008).

### Battle of the sexes

If we consider Case 1 (power-motivated) agents playing BoS, we see that *E*_1_(*C*) > *E*_1_(*D*) for *P*_2_(*C*) = 0 and *E*_1_(*D*) > *E*_1_(*C*) for *P*_2_(*C*) = 1. *E*_1_(*C*) and *E*_1_(*D*) intersect at the point:

$$P_{2}(C)=\frac{2I^{*}-S-P}{2I^{*}-S-P+T-R}$$

**Figure 9:**
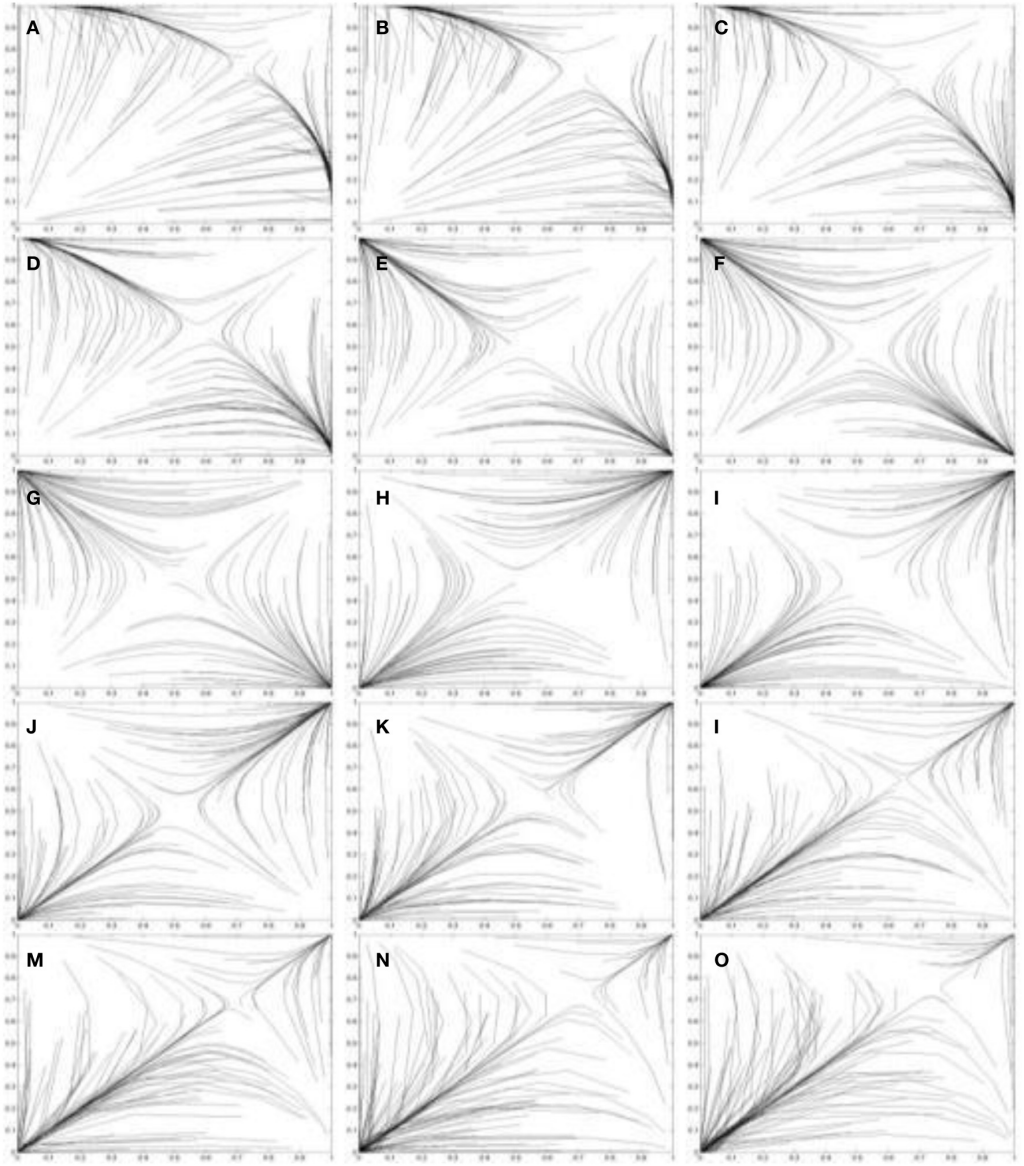
**Simulations of one hundred pairs of agents playing thirty iterations of the Battle-of-the-Sexes game**. Agents share different values of I^*^_j_ in each simulation. **(A)** I^*^_j_ = 3.8; **(B)** I^*^_j_ = 3.6; **(C)** I^*^_j_ = 3.4; **(D)** I^*^_j_ = 3.2; **(E)** I^*^_j_ = 3.0; **(F)** I^*^_j_ = 2.8; **(G)** I^*^_j_ = 2.6; **(H)** I^*^_j_ = 2.4; **(I)** I^*^_j_ = 2.2; **(J)** I^*^_j_ = 2.0; **(K)** I^*^_j_ = 1.8; **(L)** I^*^_j_ = 1.6; **(M)** I^*^_j_ = 1.4; **(N)** I^*^_j_ = 1.2; **(O)** I^*^_j_ = 1.0. Initial values of *p*_*C*_ and *q*_*C*_ are randomized. See Figure 5 for legend.

Now, suppose we have two pairs of learning agents playing a BoS game. The first pair of agents has optimally motivating incentives I^*^_1_ = I^*^_2_ = I^*^_j_. The second pair has optimally motivating incentives I^*^_1_ = I^*^_2_ = I^*^_k_ such that I^*^_j_ < I^*^_k_. This implies *P*_*j*_(*C*) < *P*_*k*_(*C*) as the (*T* − *R*) term in the denominator becomes increasingly significant as I^*^ decreases. In other words, the probability of choosing *C* decreases in agents with lower values of I^*^ as they begin to perceive the *D* choice as a desirable act of leadership rather than as a less desirable act of sacrifice. This is evident in the simulations in Figure 9. Figure 9 uses the two population replicator dynamics in Equations 1 and 2 to simulate one hundred pairs of agents (*A*_1_ and *A*_2_) playing the BoS game:

$$G=\left[\begin{array}{cc} 1 & 3\\ 4 & 2 \end{array}\right]$$

Figures 9A,B show Case 1 simulations while Figures 9C,D show Case 2 simulations in which the learning agents perceive a Leader game (Theorem 2.4.3) rather than the original BoS game. Progressively more direct trajectories towards the (*C*, *D*) and (*D*, *C*) outcomes are evident in these simulations as I^*^_j_ decreases.

Figures 9E–G show simulations in which the agents perceive a Chicken game rather than a BoS game. This is followed by another change in perception in Figures 9H,L. In these simulations, and in the Case 3 games in Figures 9M,N the perceived games have two pure NE: (*D*, *D*) and (*C*, *C*). The strategy chosen by the agents depends on the initial values of *p*_*C*_ and *q*_*C*_. These pure strategy equilibria result in both players attending entertainment alone. For the best outcome to emerge, either a “hero,” a “leader,” or a “chicken” personality is required.

### Strategic interactions between agents with different motives

**Figure 10:**
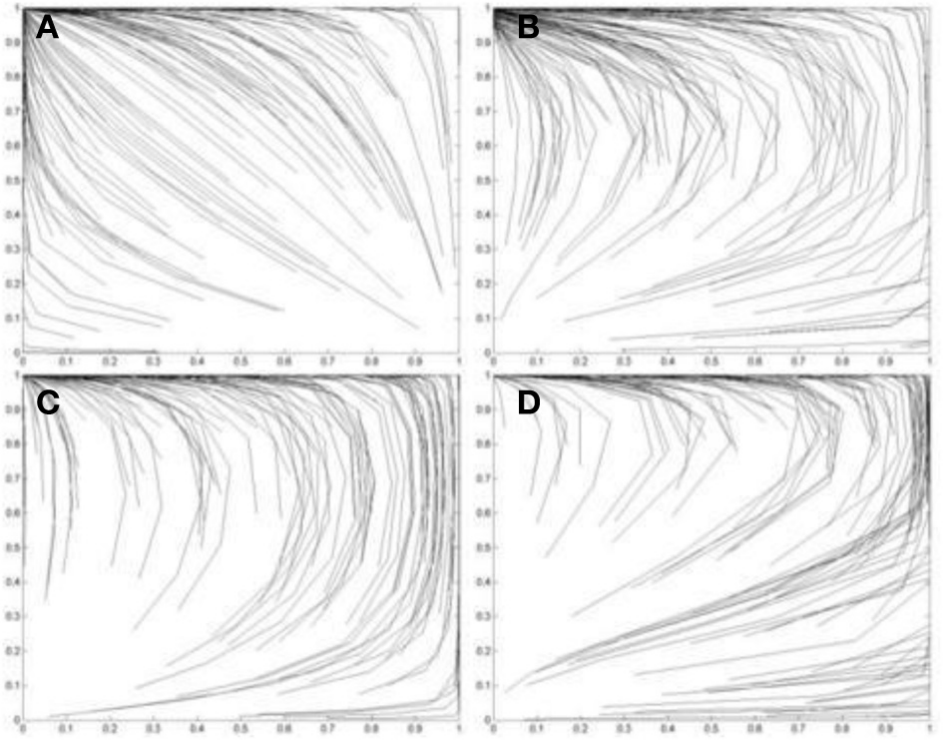
**Simulations of one hundred pairs of agents playing thirty iterations of (A) the Prisoner's Dilemma game; (B) the Leader game; (C) the Chicken game; and (D) the Battle-of-the-Sexes game**. In each simulation, one agent in each pair has I^*^_1_ = 3.9 and the other has I^*^_2_ = 1.1. Initial values of *p*_*C*_ and *q*_*C*_ are randomized. See Figure 5 for legend.

The simulations so far consider pairs of agents with the same optimally motivating incentives. However, it is also possible to simulate the outcomes when pairs of learning agents with different optimally motivating incentives interact. Figures 10A–D simulates such pairs of agents playing each of the four games, PD, Leader, Chicken, and BoS, respectively. In each pair, one agent *A*_1_ has a high optimally motivating incentive I^*^_1_ = 3.9 and the other *A*_2_ has a low optimally motivating incentive I^*^_1_ = 1.1.

The results in Figure 10 show that agents with high optimally motivating incentive tend to be the “exploiters” in PD and Chicken games, the “leaders” in a Leader game, and the “heroes” in a BoS game. In contrast, agents with low optimally motivating incentive (less than the average of the lowest two payoffs of a game) tend to be the “martyrs” in a PD game, the “followers” in a Leader game, the “chickens” in a Chicken game and the “selfish” in a BoS game.

## Discussion

In this paper we have represented agents with an optimally motivating incentive that influences the way they perceive the payoffs in strategic interactions. By using two-by-two mixed-motive games to represent different kinds of strategic interactions, we have shown that agents with different optimally motivating incentives perceive the original game differently. In many cases the perceived games have different equilibrium points to the original game. We can draw a number of general conclusions about the perceptions of agents with different optimally motivating incentives:

- Agents with high optimally motivating incentive (greater than the average of the highest two payoffs of a game) perceive a game that still conforms to the conditions defining the original game. For example, an agent with high optimally motivating incentive playing a PD game will still perceive a valid PD game and so on.
- Agents with moderate or lower optimally motivating incentive perceive new games that do not conform to the conditions defining the original game. This changes the NE and the behavior of the agents over time.

When agents with different optimally motivating incentives interact:

- Agents with high optimally motivating incentive will tend to be the “exploiters” in PD and Chicken games, the “leaders” in a Leader game, and the “heroes” in a BoS game.
- Agents with low optimally motivating incentive (less than the average of the lowest two payoffs of a game) will tend to be the “martyrs” in a PD game, the “followers” in a Leader game, the “chickens” in a Chicken game and the “selfish” in a BoS game.

The concept of optimally motivating incentive thus provides an approach to building artificial agents with different personalities using motivation. Personality in this case is expressed through behavior. For example, using the language of Colman (1982), agents in the simulations in section Results can be interpreted as demonstrating behavioral characteristics such as “aggression,” “leadership,” “heroism,” “martyrdom,” and “caution.” This suggests a number of possible applications including the design of more believable agents, human-computer interaction and simulation of human decision-making. These are discussed in the following sub-sections.

### Believable agents

Agents with distinguishable personalities have applications in areas such as animated entertainment where believable agents increase the sense of immersion in a virtual environment. According to Loyall (1997), believable agents should “*allow people to not just watch, but also interact with… powerful, personality-rich characters.*” The work in this paper specifically explores the role of intrinsic motivation for artificial agents engaged in social interactions. While the experiments in this paper are abstracted to the decision-making level, it is feasible to imagine an extension of this work in which this decision making controls the animated behaviour of a virtual character.

Some existing work has studied self-motivated behavior such as curiosity and novelty-seeking in NPCs in computer games (Merrick and Maher, 2009). Merrick and Maher (2009) demonstrate that intrinsically motivated reinforcement learning agents can learn in open-ended environments by generating goals in response to their experiences. The simulations in this paper combined optimally motivating incentive with learning using replicator dynamics, to complement the analytical description of each game transformation. However, in future it is feasible that motive profiles may be combined with learning algorithms that learn from actual interaction and experimentation with their environment during strategic interactions. Reinforcement learning variants such as frequency adjusted Q-learning (Kaisers and Tuyls, 2010) have been specifically developed for such multi-agent systems and suggest a starting point for such work. This would permit a wider range of motives to be used in NPCs. It would also extend existing work with intrinsically motivated NPCs from scenarios in which individual agents interact with their environment to scenarios in which multiple intrinsically motivated agents interact with each other.

### Human-computer interaction

Just as the study of computational models of motivation lies at the intersection of computer science and cognitive science, another area of future work lies at the boundary where computer and human interact. In particular, computers are increasingly applied to problems that require them to develop beliefs about the motives and intentions of the humans with whom they interact. Maher et al. (2007) for example, propose “*curious places*” in which a building is an “immobile robot” with sensors an actuators permitting it to monitor and control the built environment. The aim of the immobile robot is to intervene proactively on behalf of the human and modify the environment in a manner that supports the human's goals. In order to do this, it must first identify those goals.

The framework in this paper can be conceived as a foundation for agents to simulate and reason about the decision-making of other agents or humans. As discussed in section Mixed-Motive Games, the four games studied in this paper represent abstractions of real-world interaction scenarios. A robot equipped with appropriate sensors might monitor the behavior of a given human in such scenarios and deduce their motive profile from their behavior. By engaging in such “autonomous mental simulation” of the intrinsically motivated reasoning of another, such an agent may ultimately be better equipped to estimate and support the goals of humans.

### Simulation of human decision-making

The theories presented in this paper provide a starting point for developing populations of agents that can reproduce certain aspects of human decision-making during strategic interactions. Merrick and Shafi (2011) showed that it is possible to calibrate power, achievement and affiliation motivated agents such that they can accurately simulate human decision-making under certain constrained conditions. Specifically, their work focused on single-shot decisions by individual agents. The work in this paper provides a foundation for extending their work to scenarios in which agents interact. In future, such simulations may permit us to examine hypotheses about how individuals with different motives may behave during strategic interactions.

Key research challenges in this area include understanding the ranges of optimally motivating incentives that best represent motivation types such as power, affiliation and achievement motivated individuals. In practice it seems that there is significant overlap between individuals in the three groups. In addition, motivation psychologists have identified hybrid profiles where more than one motive is dominant (Heckhausen and Heckhausen, 2008). For example in the leadership profile both power and achievement motivation are believed to have approximately equal strength. In terms of the work in this paper, this would mean that agents have more than one optimally motivating incentive. Exploration of profiles such as this is a direction for future work that can provide insight into both the role of motivation in humans and its modeling in artificial systems.

### Conflict of interest statement

The authors declare that the research was conducted in the absence of any commercial or financial relationships that could be construed as a potential conflict of interest.
